# Prognostic significance of circulating immune-inflammatory biomarkers IL-6 and systemic immune-inflammation index in hepatocellular carcinoma treated with immune checkpoint inhibitors: A meta-analysis

**DOI:** 10.5937/jomb0-64710

**Published:** 2026-06-16

**Authors:** Enze Hu, Yuanfu Qiu

**Affiliations:** 1 The Sixth Hospital of Wuhan, Affiliated Hospital of Jianghan University, Department of Pharmacy, Wuhan, China; 2 Sun Yat-Sen Memorial Hospital, Department of Clinical Laboratory, Sun Yat-Sen University, Guangzhou, China

**Keywords:** immune checkpoint inhibitors, hepatocellular carcinoma, inflammatory markers, IL-6, SII, prognosis, meta-analysis, inhibitori imunoloških kontrolnih tačaka, hepatocelularni karcinom, inflamatorni markeri, IL-6, SII, prognoza, meta-analiza

## Abstract

**Background:**

Hepatocellular carcinoma (HCC) is characterized by a chronic inflammatory and immunologically dysregulated microenvironment. Circulating immune-inflammatory biomarkers reflect systemic biochemical and immune alterations and may provide laboratory-accessible indicators for prognosis assessment. However, the prognostic value of interleukin-6 (IL-6) and the systemic immune-inflammation index (SII) in HCC patients receiving immune checkpoint inhibitors (ICIs) remains incompletely defined.

**Methods:**

A comprehensive literature search was conducted across PubMed, Embase, Web of Science, the Cochrane Library, CNKI, and Wanfang Data to identify cohort studies evaluating circulating IL-6 and/or SII in HCC patients treated with ICIs. Hazard ratios (HR) with 95% confidence intervals (CI) for progression-free survival (PFS) and overall survival (OS) were pooled using fixedor random-effects models. Subgroup and sensitivity analyses were performed to explore sources of heterogeneity.

## Abstract (sr)

**Uvod:**

Hepatocelularni karcinom (HCC) karakteriše hronicno inflamatorno i imunološki disregulisano mikrookruženje. Cirkulišući imuno-inflamatorni biomarkeri odražavaju sistemske biohemijske i imunološke promene i mogu pružiti laboratorijski dostupne indikatore za procenu prognoze. Međutim, prognosticka vrednost interleukina-6 (IL-6) i sistemskog indeksa imuno-inflamacije (SII) kod pacijenata sa HCC koji primaju inhibitore imunskih kontrolnih tacaka (IKI) ostaje nepotpuno definisana.

**Metode:**

Sprovedena je sveobuhvatna pretraga literature u bazama podataka PubMed, Embase, Web of Science, Cochrane Library, CNKI i Wanfang Data kako bi se identifikovale kohortne studije koje procenjuju cirkulišući IL-6 i/ili SII kod pacijenata sa HCC lečenih ICI. Odnosi rizika (HR) sa 95% intervalima poverenja (CI) za preživljavanje bez progresije (PFS) i ukupno preživljavanje (OS) su objedinjeni korišćenjem modela sa fiksnim ili slučajnim efektima. Analize podgrupa i osetljivosti su sprovedene kako bi se istražili izvori heterogenosti.

**Rezultati:**

Uključeno je dvanaest studija koje su obuhvatile 1.508 pacijenata. Povišeni nivoi cirkulišućeg IL-6 bili su značajno povezani sa lošijim PFS (HR=1,91, 95%CI 1,40-2,60) i OS (HR=2,13, 95%CI 1,52-2,97), sa robusnim rezultatima u analizama osetljivosti. Nasuprot tome, prognosticka vrednost SII bila je zavisna od konteksta. Viši SII bio je značajno povezan sa kraćim PFS kod pacijenata sa HCC koji nisu 100% povezani sa HBV-om i sa lošijim OS kod pacijenata koji su primali kombinovani lokalni tretman, dok u drugim podgrupama nisu primećene značajne povezanosti.

**Zaključak:**

Cirkulišući IL-6 služi kao stabilan imuno-biohemijski prognostički biomarker kod pacijenata sa HCC lečenih ICI, dok je prognostički značaj SII moduliran etiologijom bolesti i načinom lečenja. Ovi nalazi pružaju laboratorijski relevantne biohemijske dokaze koji podržavaju integraciju cirkulišućih imuno-inflamatornih markera u prognostičke okvire za procenu HCC.

## Introduction

Hepatocellular carcinoma (HCC) accounts for 90% of primary liver cancer diagnoses and ranks as the fourth leading cause of cancer-related deaths globally [1]. For patients diagnosed at an early stage, therapeutic options such as surgical resection, ablation therapy, transarterial chemoembolization, and liver transplantation yield significant efficacy, with some achieving clinical cure. However, HCC typically presents no obvious symptoms, and most cases are diagnosed at an advanced stage, resulting in the loss of opportunities for curative surgery; thus, systemic therapy has become the mainstay of treatment [2]. In recent years, immune checkpoint inhibitors (ICIs) represented by nivolumab and pembrolizumab have broken the »monopoly« of anti-angiogenic targeted drugs by blocking inhibitory signals between tumor cells and the immune system, activating the patient's intrinsic immune response to attack and suppress tumor growth, ultimately leading to a significant extension of survival [3]. Over 90% of HCC patients have a history of chronic hepatitis, and inflammation promotes HCC progression by enhancing tumor cell proliferation and survival signals, inducing angiogenesis, facilitating immune escape, and initiating invasion and metastasis [4]. Among inflammatory mediators, interleukin-6 (IL-6) serves as a key driver in the development of HCC and other tumors [5]. Meanwhile, the systemic immune-inflammation index (SII), a comprehensive inflammatory marker integrating neutrophil, platelet, and lymphocyte counts, has been shown that patients with high SII levels are more prone to microvascular invasion compared to those with low SII [6]. Therefore, the occurrence and progression of HCC are closely associated with a chronic inflammatory microenvironment, making the regulation of inflammatory responses a potential crucial intervention target for improving patient prognosis. Previous meta-analyses have indicated that inflammatory biomarkers such as C-reactive protein and neutrophil-to-lymphocyte ratio can predict the prognosis of HCC patients treated with ICIs [7], However, the prognostic value of IL-6 and SII in HCC patients receiving ICIs has not been systematically validated through meta-analysis, and their utility as non-invasive biomarkers for evaluating ICI efficacy remains unclear. Thus, this study aims to conduct a Meta-analysis to assess whether IL-6 and SII can predict the prognosis of HCC patients treated with ICIs.

## Materials and methods

### Literature retrieval strategy

Databases including China National Knowledge Infrastructure (CNKI), Chinese Biology Medicine , Wanfang Data, PubMed, Embase, The Cochrane Library, and Web of Science were searched from January 1997 to January 2026. A combination of subject terms and free-text words was used for retrieval. Chinese and English search terms: hepatocellular carcinoma, immune checkpoint inhibitors, Nivolumab, Pembrolizumab, Atezolizumab, Durvalumab, Sintilimab, Camrelizumab, IL-6, SII, inflammation, PFS, OS et al. Search Formula for PubMed: (hepatocellular carcinoma[Title/Abstract] OR HCC[Title/Abstract]) AND (immune checkpoint inhibitors[Title/Abstract] OR ICIs[Title/Abstract] OR Nivolumab[Title/Abstract] OR Pembrolizumab[Title/Abstract] OR Tremelimumab[Title/Abstract]) OR Durvalumab[Title/Abstract] OR Camrelizumab[Title/Abstract] OR Sintilimab[Title/Abstract]) AND (Interleukin-6[Title/Abstract] OR IL-6[Title/Abstract] OR S11 [Title/Abstract] OR Immune-Inflammation Index[Title/Abstract] OR Inflammation[Title/Abstract] AND (PFS[Title/Abstract] OR OS[Title/Abstract] OR progression-free survival[Title/Abstract] OR overall survival[Title/Abstract].

### Inclusion and exclusion criteria

Inclusion Criteria: (1) Study subjects were patients diagnosed with HCC; (2) Study types were publicly published prospective cohort studies or retrospective cohort studies; (3) Intervention was treatment with ICIs; (4) Outcome measures included at least one of the following indicators after treatment: PFS, OS, IL-6, or SII; (5) Articles providing available or calculable hazard ratios (HR) with 95% confidence intervals (95% CI).

Exclusion Criteria: (1) Duplicate published literature; (2) Conference abstracts, reviews, systematic analyses, animal experiments, case reports, or master's theses; (3) Study subjects with other types of liver cancer or concurrent diseases such as sarcopenia; (4) Unavailable full-text articles; (5) Literature with poor quality or unclear data; (6) Literature not written in Chinese or English.

### Literature screening and data extraction

Two reviewers independently screened the literature, extracted data, and cross-validated the results. In case of discrepancies, a third reviewer was consulted for arbitration. Missing data were attempted to be supplemented by contacting the corresponding authors. The following data were extracted: first author's name, year of publication, sample size, age of patients, study type, type of inflammatory biomarkers, drug treatment regimens, and survival outcomes (e.g., progression-free survival and overall survival). All HR and 95% CI extracted in this study were derived from multivariable-adjusted models in the original studies.

### Quality assessment

Two reviewers independently evaluated the methodological quality of prospective cohort studies using the Newcastle-Ottawa Scale (NOS), which includes three domains: patient selection, comparability of study groups, and outcome assessment. Each study was scored on a scale of 0 to 9 points: studies with a score of ≥7 were considered high quality, those with 5-6 points were moderate quality, and those with ≤4 points were low quality.

### Statistical analysis

Meta-analysis was performed using RevMan 5.3 software and Stata Mp HR with 95% CI were used as effect size indicators for count data. Heterogeneity among studies was evaluated by the Q test and I^2^ statistic. If I^2^ ≤ 50% and P ≥ 0.10, no statistical heterogeneity was considered to exist, and a fixed-effects model was adopted; otherwise, a random-effects model was used, and sensitivity analysis was conducted via the stepwise exclusion method to assess the impact of each individual study on the overall results, thereby verifying the robustness of the Meta-analysis findings. Subgroup analyses were prespecified before data synthesis. HBV status and local treatment were selected as subgroup variables based on previous clinical evidence and clinical rationales, aiming to explore potential sources of heterogeneity and avoid post hoc analysis. The significance level was set at a = 0.05.

## Results

### Literature search and screening

**Figure 1:**
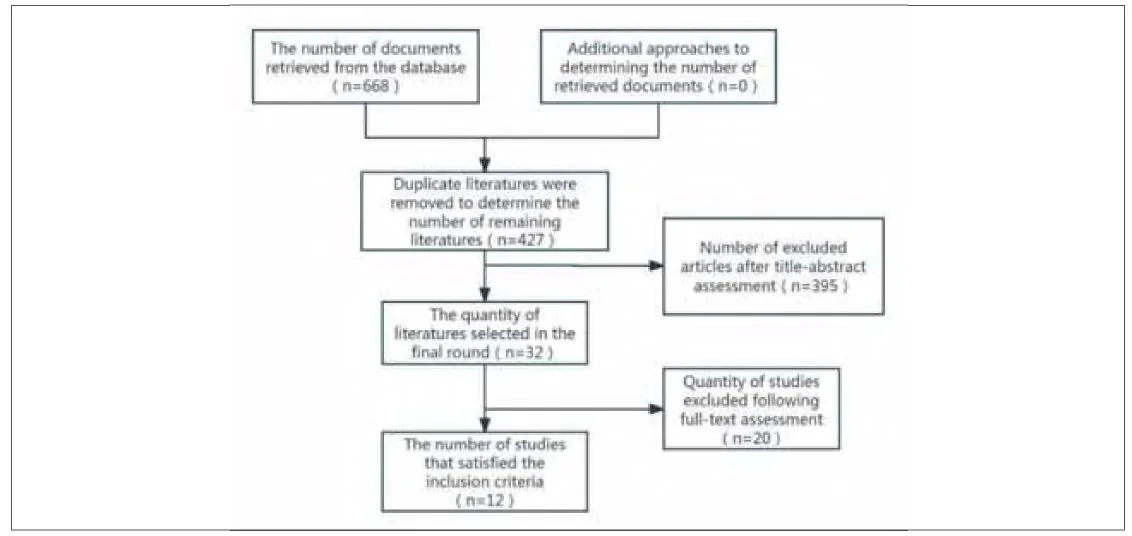
Literature retrieval flow chart.

A total of 668 articles were obtained from the first search. After screening by inclusion and exclusion criteria, 12 articles met the inclusion criteria of this meta-analysis. The process of research screening is shown in Figure 1.

### Basic characteristics of the included studies

A total of 12 retrospective observational studies were finally included in this meta-analysis, among which 10 were retrospective cohort studies. All of these cohort studies were conducted based on fixed patient cohorts to analyze the association between exposures and outcomes. The remaining 2 were retrospective non-cohort observational studies, which did not adopt a strict cohort design and only involved retrospective summarization and correlation analysis of clinical data. The basic characteristics of the included studies are summarized in Table 1 [8][9][10][11][12][13][14][15][16][17][18][19].

**Table 1:** Characteristics of patients included in the study.

Reference	Country/<br>region	N	Age	Male/<br>female	Study<br>design	Types of<br>inflammatory<br>markers	drug treatment<br>project	Survival<br>prognostic<br>indicators	NOS<br>point	Cut-off<br>values
LIU C 2023<br>[8]	China	151	57.41<br>(9.14)	124/27	retrospective<br>cohort study	SII	atezolizumab<br>combined with<br>bevacizumab t OR<br>camrelizumab combined<br>with apatinib	PFS, OS	8	377.03
JIA G 2023<br>[9]	China	117	79.5%<br>≤65	106/11	retrospective<br>cohort study	SII	anti-PD-1 antibody	PFS, OS	8	509
DU J 2024<br>[10]	China	222	53.90±<br>11.28	189/33	retrospective<br>cohort study	IL-6	ICIs	PFS, OS	8	19.82
HUANG R<br>2022 [11]	China	110	54.5<br>(31-84)	100/10	retrospective<br>cohort study	SII	sorafenib or lenvatinib<br>treatment combined<br>with more<br>than two cycles of<br>anti-PD-1 therapy	PFS, OS	8	970
MIURA R<br>2025 [12]	Japan	90	-	-	retrospective<br>cohort study	IL-6	Atezo+Bev therapy	PFS	8	9.2 pg/<br>mL
SUZUKI T<br>2024 [13]		94	73<br>(66-78)	74/20	retrospective<br>non-cohort<br>observational<br>study	IL-6	Atez + Bev	PFS, OS	6	7.4 pg/<br>mL
YAO Y 2025<br>[14]	China	117	76.9%<br><65	92/25	retrospective<br>cohort study	SII	lenvatinib in combination<br>with a PD-1<br>inhibitor (Camrelizumab,<br>Tislelizumab,<br>Cedilimumab)	PFS, OS	6	539.47<br>or<br>303.66
MYOJIN Y<br>2022 [15]	Japan	64	75<br>(63, 79)	50/14	retrospective<br>cohort study	IL-6	Atezo/Bev therapy	PFS	6	4.77
KOCHEISE L<br>2026 [16]	Europe	94	73<br>(66-78)	74/20	retrospective<br>cohort study	IL-6	Atez+Bev	OS	8	18.22<br>pg/mL
ZHANG T<br>2024 [17]	China	189	76.7%<br><60	169/20	retrospective<br>non-cohort<br>observational<br>study	SII	camrelizumab<br>+tyrosine<br>kinaseinhibitors	OS	6	-
ZHUANG M<br>T 2024 [18]	China	140	51.43%<br><60	114/26	retrospective<br>cohort study	SII	TACE+camrelizum-ab<br>+tyrosine kinase<br>inhibitors	PFS, OS	6	303.09
JIANG S<br>2023 [19]	China	120	57.5±<br>9.8	99/21	retrospective<br>cohort study	SII	Immunotherapy<br>(Caprelizumab,<br>Paporizumab, Tirelizumab,<br>Sindilizumab)<br>+ targeted<br>therapy	OS	6	626.21

### Quality assessment

A total of 12 studies were included in this Meta-analysis, and the methodological quality of all included studies was evaluated using the NOS. Among them, 6 studies were rated as high quality, and 6 were moderate quality (Table 1).

### Meta-analysis

### Association between SII and PFS

**Figure 2:**
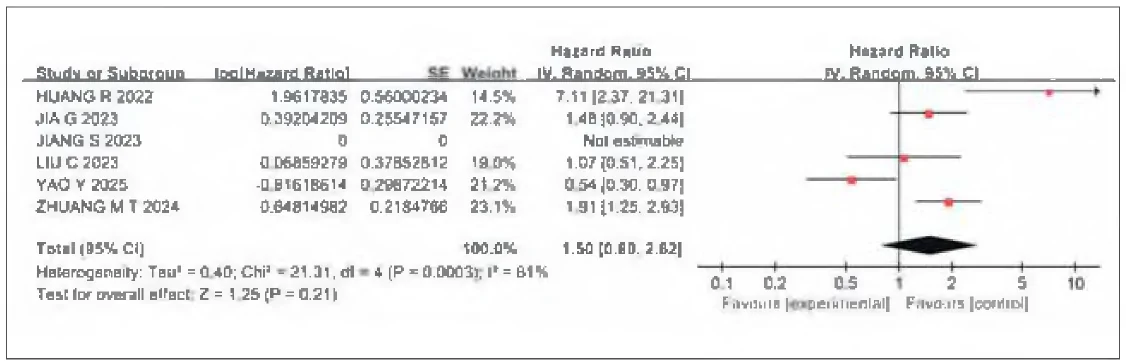
Forest plot of the correlation between SII and PFS.

Six studies reported the correlation between SII levels and PFS, with high heterogeneity observed among studies (I^2^=81 %,P<0.001 ); thus, a random-effects model was employed for Meta-analysis. The results showed that elevated SII levels were not significantly associated with PFS (HR=1.50,95%CI = 0.80~2.82;P=0.21; Figure 2).

**Figure 3:**
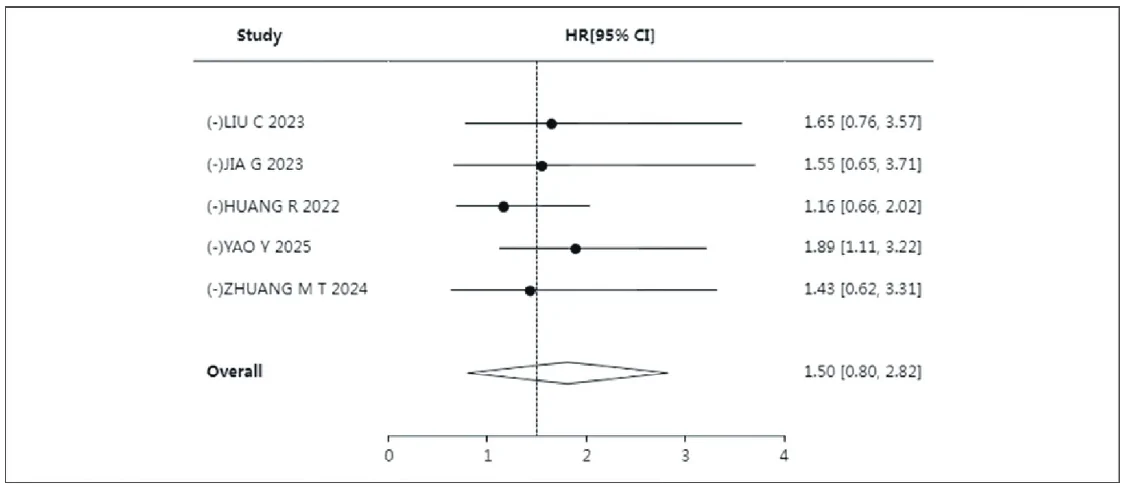
Sensitivity analysis of the correlation between SII and PFS.

To verify the stability of the results, further sensitivity analysis was performed, which revealed that the pooled result was highly sensitive to the study by Yao Y [14] after excluding this study, the pooled result changed from »no significant association« to »significant association«. In contrast, after excluding the other four studies, the pooled HR and 95%CI showed minimal fluctuations with no significant associations, indicating that these studies had limited impact on the stability of the results (Figure 3).

Combined with the sensitivity analysis results, literature review identified the core source of heterogeneity as differences in population etiology: two studies Huang R [11] Yao Y [14] included patients with 100% HBV-related HCC, while the remaining four studies included HCC patients with mixed etiologies (including non-HBV-related cases). Based on this, subgroup analysis was conducted according to »whether the population was 100% HBV-related HCC«: Subgroup of 100% HBV-related HCC (2 studies): Extremely high heterogeneity persisted among studies (I^2^ = 94%,P<0.001), and the pooled result showed no significant correlation between elevated SII levels and PFS (HR=0.95,95%CI=0.57~1.59;P=0.85); Subgroup of non-100% HBV-related HCC (4 studies): Heterogeneity among studies was completely eliminated (I^2^ = 0,P=0.39), and the pooled result indicated that elevated SII levels were significantly associated with poorer PFS in patients (HR=1.59,95%CI = 1.18~2.14; P=0.002).

### Association between SII and OS

**Figure 4:**
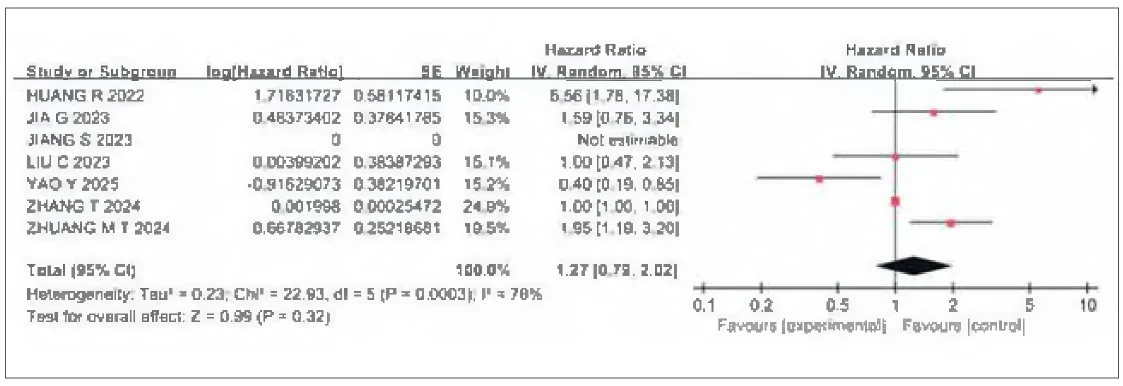
Forest plot of the correlation between SII and OS.

Seven studies reported the relationship between SII levels and OS, with high heterogeneity among studies (I^2^ = 78%,P<0.001); therefore, a random-effects model was used for Meta-analysis. The results showed no significant correlation between elevated SII levels and OS (HR=1.27, 95%CI = 0.79~2.02; P=0.32; Figure 4).

**Figure 5:**
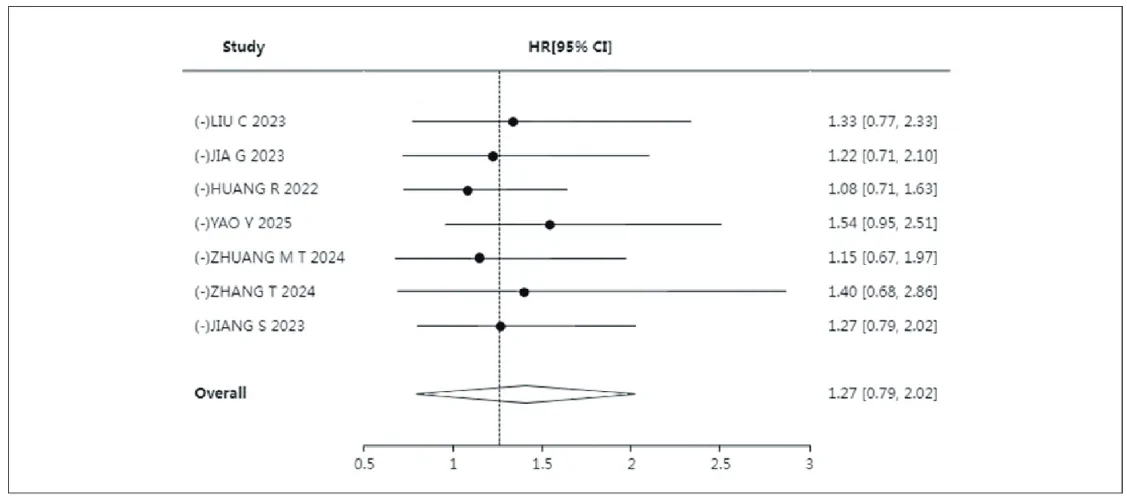
Sensitivity analysis of the correlation between SII and OS.

To verify the stability of the results, further sensitivity analysis was performed. The findings indicated that the pooled HR and 95%CI did not fluctuate significantly regardless of which study was excluded, suggesting the pooled result was relatively robust (Figure 5).

Subgroup analysis was conducted based on »whether the population was 100% HBV-related HCC«: Subgroup of 100% HBV-related HCC (2 studies): Extremely high heterogeneity remained among studies (I^2^ = 93%,P<0.001), and the pooled result showed no significant correlation between elevated SII levels and OS (HR=1.44,95%CI=0.11~18.96; P=0.78); Subgroup of non-100% HBV-related HCC (4 studies): Significant heterogeneity persisted among studies (I^2^=65%,P=0.04), and the pooled result indicated no significant correlation between elevated SII levels and OS (HR=1.28, 95%CI=0.72~2.27; P=0.21).

Another subgroup analysis was performed according to treatment strategy (»whether local treatment was administered«): Subgroup with local treatment (5 studies): Heterogeneity was reduced to 52% (P = 0.10), and the pooled result showed that elevated SII levels were significantly associated with poorer OS (HR=1.84, 95%CI = 1.08~3.14; P=0.03); Subgroup without local treatment (2 studies): High heterogeneity remained among studies (I^2^=83%, P = 0.02), and the pooled result showed no significant correlation between elevated SII levels and OS (HR=0.69, 95%CI=0.28~1.66; P=0.40).

### Association between IL-6 and PFS

**Figure 6:**
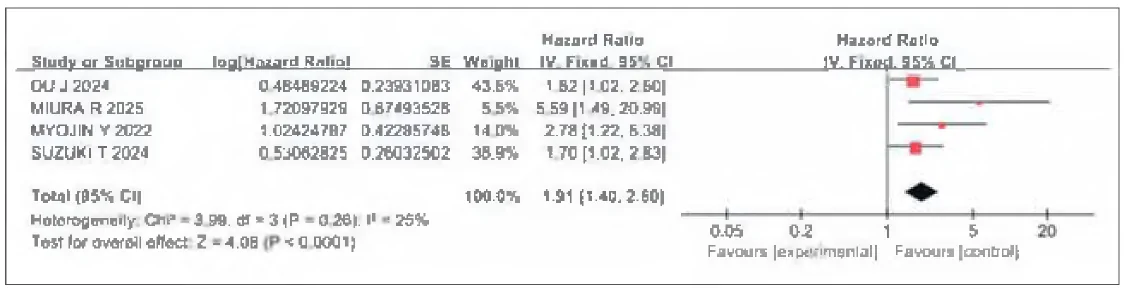
Forest plot of the correlation between IL-6 and PFS.

**Figure 7:**
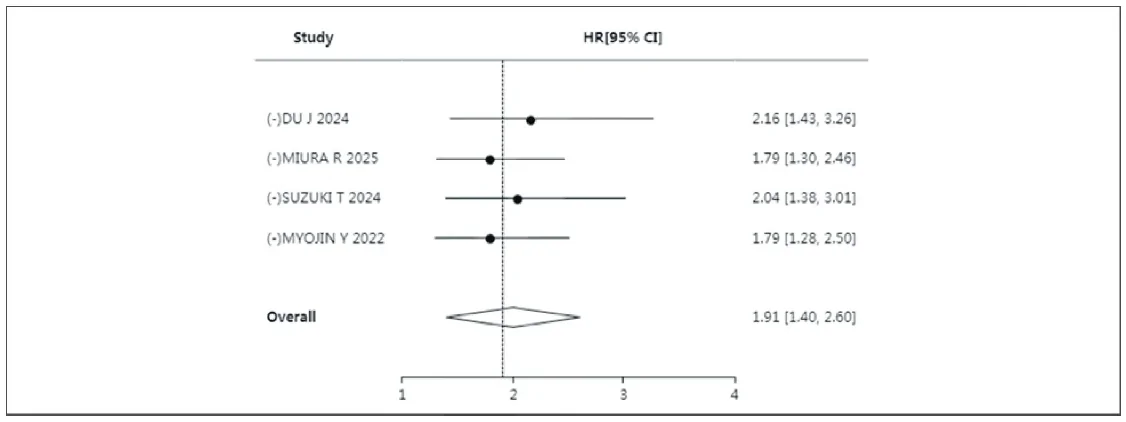
Sensitivity analysis of the correlation between IL-6 and PFS.

Four studies reported the relationship between IL-6 levels and PFS, with no substantial heterogeneity identified among the studies (I^2^=25%, P=0.26). Therefore, a fixed-effects model was used for the meta-analysis. The results showed that elevated IL-6 levels were significantly associated with poorer PFS (HR=1.91, 95%CI = 1.40-2.60; P<0.001; Figure 6). Sensitivity analysis revealed no significant fluctuations in the pooled HR and 95%CI after the exclusion of any single study, indicating that the pooled results were robust (Figure 7).

### Association between IL-6 and OS

**Figure 8:**
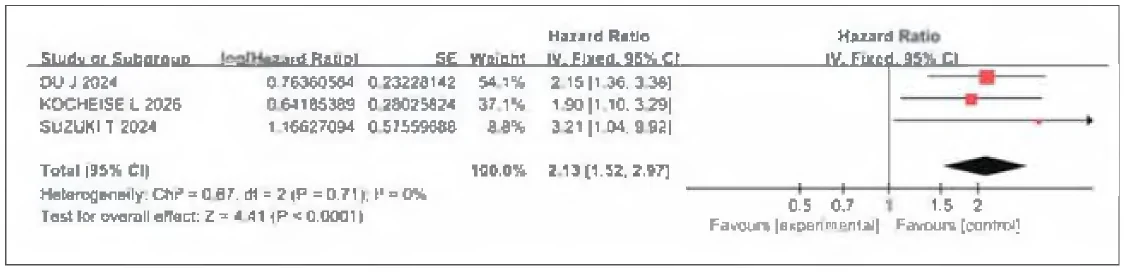
Forest plot of the correlation between IL-6 and OS.

**Figure 9:**
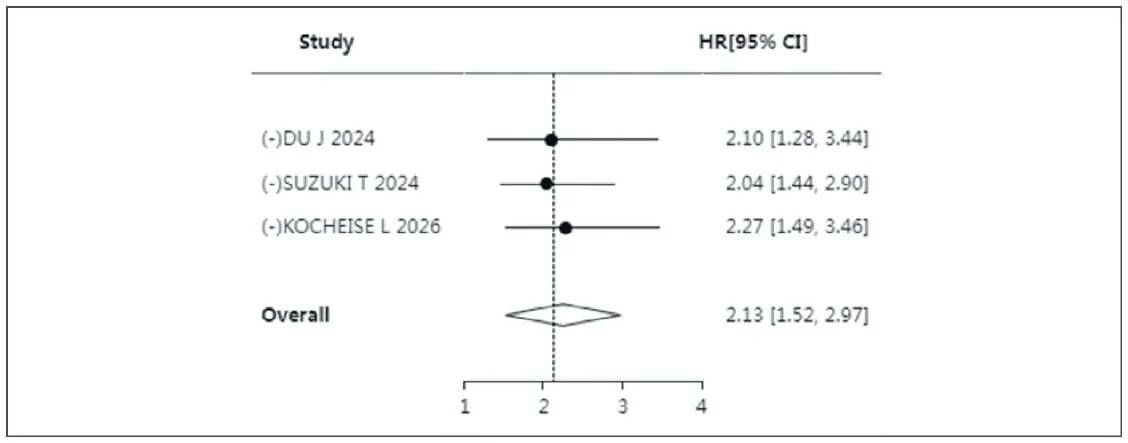
Sensitivity analysis of the correlation between IL-6 and OS.

Three studies reported the relationship between IL-6 levels and OS, with no substantial heterogeneity detected among the studies (I^2^=0%, P=0.71). A fixed-effects model was therefore applied for the meta-analysis. The results indicated that elevated IL-6 levels were significantly associated with poorer OS (HR=2.13, 95%CI = 1.52-2.97; P<0.001; Figure 8). Sensitivity analysis showed no significant fluctuations in the pooled HR and 95% CI following the exclusion of any single study, demonstrating the robustness of the pooled results (Figure 9).

## Discussion

Our Meta-analysis revealed high heterogeneity across the 6 studies investigating the association between SI I and PFS and the 7 studies exploring the relationship between SII and OS, with no significant correlation observed between elevated SII levels and either PFS or OS in HCC patients. Subgroup and sensitivity analyses were therefore performed to identify the sources of such heterogeneity. Sensitivity analysis indicated that the pooled results for the SII-PFS association were highly sensitive to previous evidence. Subgroup analysis further demonstrated that elevated SII levels were significantly associated with poorer PFS in the non-100% HBV-related HCC subgroup. In contrast, the pooled results for the SII-OS association were robust; elevated SII levels were significantly correlated with poorer OS in the subgroup of patients who received local treatment, whereas no significant association was found in the subgroup without local treatment. These findings suggest that the observed heterogeneity may be associated with etiological background and receipt of local treatment.

A Meta-analysis by Tian B et al. [20] reported a significant correlation between high SII levels and both OS and PFS in cancer patients treated with ICIs, which is inconsistent with the results of our study. The potential reasons for this discrepancy may include the following: the limited number of studies included in our analysis, which resulted in insufficient statistical power; the inclusion of multiple solid tumors in the study by TIAN B W et al [20], whereas HCC patients are mostly complicated with underlying diseases such as chronic hepatitis and liver cirrhosis, leading to an inherently distinct inflammatory microenvironment compared with other tumors; and the lack of a unified optimal cutoff value for SII across different studies. Additionally, our Meta-analysis showed that elevated IL-6 levels were significantly associated with both poorer PFS and poorer OS in HCC patients. Sensitivity analysis confirmed the robustness of these results.

From a biological mechanistic perspective, SII, a comprehensive inflammatory index integrating neutrophil, lymphocyte, and platelet counts, may be closely associated with tumor progression. Neutrophils, as inflammatory cells, can migrate into the tumor microenvironment and secrete various pro-angiogenic factors, which may provide a favorable condition for tumor growth [21]. Lymphocytes are key effector cells of the body's antitumor immunity. Previous studies have suggested that decreased lymphocyte count may be associated with shorter survival [22]. During the early stages of tumori-genesis, platelets may participate in the process of tumor cell entry into the circulation. Platelets and platelet-derived microparticles can transfer the surface adhesion receptor CD61 to tumor cells, which may further enhance the ability of tumor cells to adhere to vascular endothelial cells [23]. Therefore, elevated SII levels may partly reflect an imbalance in the tumor microenvironment characterized by enhanced inflammatory response, weakened antitumor immunity, and increased metastatic potential, suggesting that tumor cells may be more prone to growth and metastasis.Furthermore, higher SII levels may be accompanied by a reduction in peripheral immune killer cells, which may contribute to the formation of an immunosuppressive tumor microenvironment, manifested by insufficient infiltration of CD8+ T cells and natural killer cells, thereby potentially placing the body in an immunosuppressive state [24]. Interactions between IL-6 and the PD-1/PD-L1 pathway may exist. Previous studies have suggested that IL-6 may regulate PD-1 expression in monocytes and macrophages via relevant signaling pathways during hepatocarcinogenesis [25]. Moreover, anti-IL-6 neutralizing antibodies and anti-PD-1/PD-L1 antibodies may act synergistically in the tumor microenvironment, contributing to breaking immune tolerance and inhibiting tumor growth [26]. Based on the above mechanistic inferences, ICIs may exert favorable effects on the prognosis of HCC patients by alleviating the immunosuppressive status and reducing IL-6 and SII levels. However, these mechanisms still require further validation in basic and clinical studies.

This study has several limitations. First, given the high heterogeneity observed for some indicators, certain results need to be interpreted with caution. Additionally, the limited number of included studies precluded the investigation of heterogeneity sources via meta-regression, which restricts the generalizability of the conclusions. Second, marked variations in the cutoff values of SII and IL-6 across the included studies may lead to limited comparability among effect size estimates, thereby increasing between-study heterogeneity, reducing the reliability of the pooled results, and potentially affecting the direction and magnitude of the final pooled effect estimates. Therefore, caution is warranted in the interpretation of the results. Third, all included studies are retrospective, which may compromise statistical power and cannot eliminate publication bias. However, bias assessment was not performed due to the fact that fewer than 10 studies were included for each indicator. Finally, the number of studies includReferences ed for IL-6 analysis was relatively small (only 3-4 studies), which significantly limited the robustness and reliability of the pooled results. Therefore, the clinical applicability and prognostic value of IL-6 should be interpreted with great caution, and further large-scale and high-quality studies are warranted to verify the present findings.

## Conclusion

IL-6 was significantly associated with the prognosis of (HCC patients treated with ICIs, whereas the associations of the SII with PFS and OS in HCC patients were modulated by etiology (HBV-related status) and treatment modality (combination with local treatment). These findings can provide a reference for clinical individualized prognostic assessment and treatment decision-making for HCC patients receiving ICIs. Although this study has clearly established the correlation between IL-6 and the prognosis of HCC patients undergoing immunotherapy, to further enhance its clinical translational potential, it is recommended to implement standardized sampling at key treatment nodes and conduct full-course dynamic monitoring to avoid the limitation that single-time-point detection cannot capture treatment-related fluctuations in IL-6 levels. Meanwhile, standardized detection methods and reference standards should be adopted to reduce inter-laboratory systematic errors, thereby providing more consistent and reliable data support for clinical decision-making.

## Dodatak

### Acknowledgments

This study did not receive any funding in any form.

### Conflict of interest statement

All the authors declare that they have no conflict of interest in this work.
